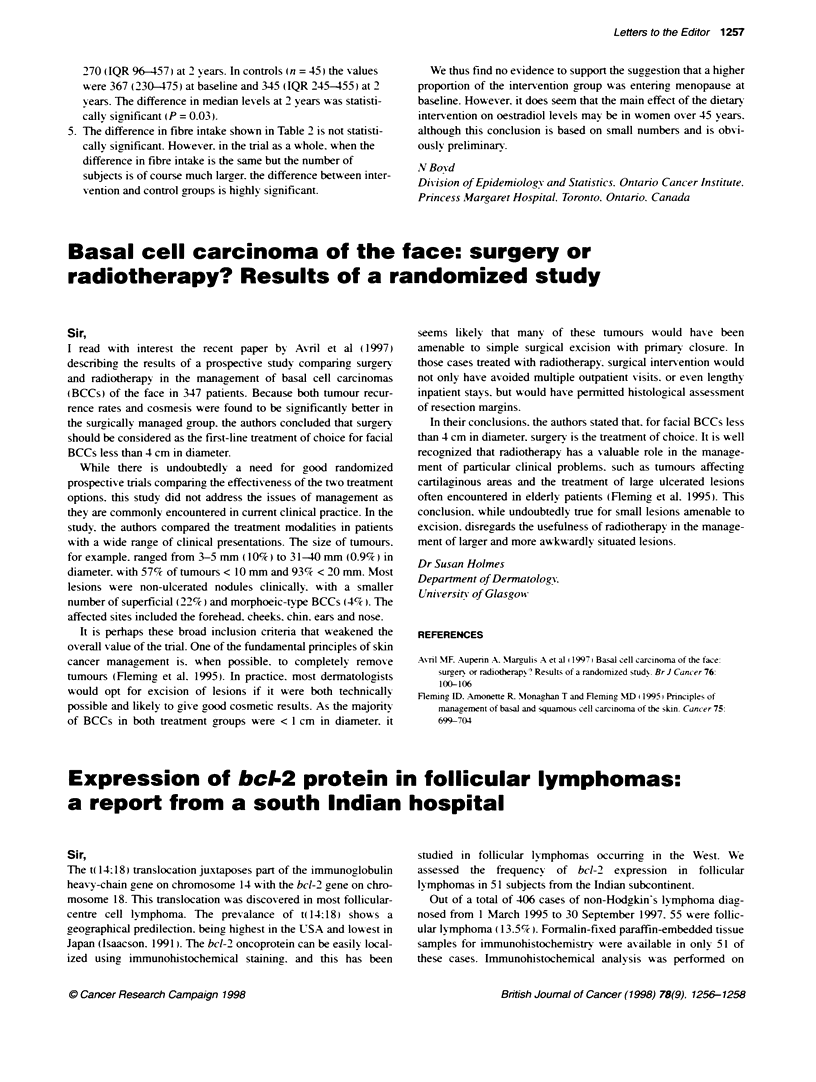# Basal cell carcinoma of the face: surgery or radiotherapy? Results of a randomized study.

**DOI:** 10.1038/bjc.1998.666

**Published:** 1998-11

**Authors:** S. Holmes


### Basal cell carcinoma of the face: surgery or radiotherapy? Results of a randomized study

**Sir,**

I read with interest the recent paper by Avril et al (1997) describing the results of a prospective study comparing surgery and radiotherapy in the management of basal cell carcinomas (BCCs) of the face in 347 patients. Because both tumour recurrence rates and cosmesis were found to be significantly better in the surgically managed group, the authors concluded that surgery should be considered as the first-line treatment of choice for facial BCCs less than 4 cm in diameter.

While there is undoubtedly a need for good randomized prospective trials comparing the effectiveness of the two treatment options, this study did not address the issues of management as they are commonly encountered in current clinical practice. In the study, the authors compared the treatment modalities in patients with a wide range of clinical presentations. The size of tumours, for example, ranged from 3–5 mm (10%) to 31–40 mm (0.9%) in diameter, with 57% of tumours < 10 mm and 93% < 20 mm. Most lesions were non-ulcerated nodules clinically, with a smaller number of superficial (22%) and morphoeic-type BCCs (4%). The affected sites included the forehead, cheeks, chin, ears and nose.

It is perhaps these broad inclusion criteria that weakened the overall value of the trial. One of the fundamental principles of skin cancer management is, when possible, to completely remove tumours (Fleming et al, 1995). In practice, most dermatologists would opt for excision of lesions if it were both technically possible and likely to give good cosmetic results. As the majority of BCCs in both treatment groups were < 1 cm in diameter, it seems likely that many of these tumours would have been amenable to simple surgical excision with primary closure. In those cases treated with radiotherapy, surgical intervention would not only have avoided multiple outpatient visits, or even lengthy inpatient stays, but would have permitted histological assessment of resection margins.

In their conclusions, the authors stated that, for facial BCCs less than 4 cm in diameter, surgery is the treatment of choice. It is well recognized that radiotherapy has a valuable role in the management of particular clinical problems, such as tumours affecting cartilaginous areas and the treatment of large ulcerated lesions often encountered in elderly patients (Fleming et al, 1995). This conclusion, while undoubtedly true for small lesions amenable to excision, disregards the usefulness of radiotherapy in the management of larger and more awkwardly situated lesions.

*Dr Susan Holmes*
*Department of Dermatology,*
*University of Glasgow*

### REFERENCES

Avril MF, Auperin A, Margulis A et al (1997) Basal cell carcinoma of the face: surgery or radiotherapy? Results of a randomized study. *Br J Cancer* **76**: 100–106

Fleming ID, Amonette R, Monaghan T and Fleming MD (1995) Principles of management of basal and squamous cell carcinoma of the skin. *Cancer* **75**: 699–704